# Editorial: Nutritional Management for the Energy Metabolism in Animals

**DOI:** 10.3389/fvets.2022.900736

**Published:** 2022-07-28

**Authors:** Jing Wang, Tarique Hussain, Yehui Duan

**Affiliations:** ^1^Hunan Provincial Key Laboratory of Animal Nutritional Physiology and Metabolic Process, College of Animal Science and Technology, Hunan Agricultural University, Changsha, China; ^2^Animal Sciences Division, Nuclear Institutes for Agriculture and Biology, Faisalabad, Pakistan; ^3^Institute of Subtropical Agriculture, Chinese Academy of Sciences (CAS), Changsha, China

**Keywords:** energy metabolism, energy disorder, nutritional management, microbiome, mitochondria

Energy metabolism is the process of generating energy (ATP) from nutrients, including both aerobic respiration (oxygen present), anaerobic respiration (fermentation) as well as fatty acid and amino acid metabolism (1). Energy homeostasis is central to animals to maintain normal function and production.

The Research Topic includes 14 papers, one review, and 13 research articles, concerning energy metabolism and its nutritional management, as well as the effect of gut microbiome and host mitochondria on energy homeostasis at various environmental situations and physiological stages of animals.

One study, Du et al. investigate the effect of different dietary energy levels on the rumen bacteria and meat quality in yak. They suggest that muscle quality of longissimus pectoris of yak fed with high dietary energy level was better, evidenced by the significantly increased water content and crude fat content. Additionally, the high energy diet also elevated the abundance of bacteria related to carbohydrate metabolism in the rumen. They draw the conclusion that high energy diet improved the meat quality of yak mainly by affecting the ruminal amyl lytic bacteria abundance to provide substrates for fatty acid synthesis.

Animals adapt to various changing environment by adjusting their development, metabolism and behavior to improve their chances of survival and reproduction (2). In this Research Topic, Kong et al. use the metabonomics and blood biochemical indexes to investigate the metabolic changes of dairy cows in different high-altitudes. With the increasing of altitude, the different metabolites are mainly enriched in amino acid metabolism and sphingolipid metabolism. And sphingolipid metabolism showed a negative correlation with increased altitude. Meanwhile, they (Kong et al.) uncover the specific mammary metabolic mechanism in hypoxic dairy cows. The results reveal that hypoxia exposure was associated with the elevation of AGPAT2-mediated glycerophospholipid metabolism. These intracellular metabolic disorders consequently lead to the lipid disorders associated with apoptosis of bovine mammary epithelial cells. A certain key nutrient deficiency also affects the metabolism and reproduction. A study of Qian et al. shows that vitamin E deficiency in the early post-partum period of cow will significantly down-regulate the apolipoprotein A3, serum amyloid protein A4 and pantetheinase-1 protein abundance in plasma, among which pantetheinase-1 is closely related to dairy cow subclinical vitamin E deficiency and can be a potential biomarker.

Natural materials of animal origin are involved in regulating nutrient metabolism (3, 4). The paper by Mesgaran et al. indicate that rumen-protected l-carnitine plays a role in supporting production, enhancing liver metabolism and regulating health biomarkers of high-yielding dairy cows during perinatal period. Based on these findings, the authors suggest that the effects of perinatal feeding of l-carnitine on the uterus should be further studied to determine its impact on offspring performance and health. Wang X. et al. find that estrogen promotes glycogen synthesis and storage, and maintains energy homeostasis by enhancing extracellular glucose uptake and regulating autophagy. They suggest that estrogen is necessary to protect cells from apoptosis and enhance the immune potential of PMNs. However, the mechanism by which estrogen regulates glucose metabolism remains unclear.

In addition to animal derived natural materials, plant extracts and probiotics also show the strong ability to interfere the animal nutritional metabolism. Four studies about plant extracts are presented in this Research Topic. Based on the beneficial effect of resveratrol on intestinal injury, the authors (Huang et al.) find that resveratrol can improve intestinal injury induced by deoxynivalenol through mitochondrial autophagy in weaned piglets. Yang et al. find that dietary supplementation of *Eucommia ulmoides* leaf extract (ELE) in finishing pigs improves the carcass traits and reduces the lipid levels by activating the AMPK-ACC pathway to regulate lipid metabolism. Results from Wang Q. et al. indicate that feeding *Phragmites australis* shoot remainder (PSR) silage could improve the growth performance, alter the rumen bacteria diversity and the corresponding function. They demonstrate that PSR silage could partially substitute (30%) corn silage for beef cattle breeding. The study of Afzal et al. elaborates that dietary supplementing with 3.5% *Moringa oleifera* leaf powder (MOLP) improves the antioxidant status, milk yield, and reproductive performance in goats. Moreover, Han et al. suggest that maternal dietary supplementation with *Bacillus subtilis* protease and *Bacillus subtilis* improves the reproductive performance and overall health indicators of sows, as well as the growth and development of their offspring.

In recent years, the nutrition and metabolism of special economic animals and pets have also attracted much attention. Bao et al. find that metabolizable energy intake (MEI), caloric production (HP) and retained energy (RE) of male Sika deer decreased significantly as the apparent digestibility of carbon and nitrogen increased with the decrease of feed intake. Particularly, they calculate the net N requirement for maintenance (NNm) and net protein requirement for maintenance (NPm) of growing male sika deer, fill the gap in net energy and protein requirements and serve as basic data for establishing the nutritional standards for sika deer breeding in China. Obesity is troubling the health of pet dogs. Lyu et al. compare the inflammatory response and fecal metabolome of dogs fed a high-fat vs. a high-starch diet, and suggests a high-starch diet is more suitable for feeding pet dogs. The high-starch diet promotes the lipid metabolism, antioxidant effects, protein biosynthesis and catabolism, mucosal barrier function and immune regulation compared to a high-fat diet in healthy lean dogs. Additionally, Li has conducted an interesting review on a state-of-the-art overview on recent advances in systems biology in canine cardiac disease. He discusses this topic based on three aspects: (1) the changes occurring in each of the three components of energy metabolism in myxomatous mitral valve disease (MMVD) and heart failure (HF); (2) the changes in circulating and myocardial glutathione, taurine, carnitines, branched-chain amino acid catabolism and tryptophan metabolic pathways; (3) the potential role of the gut microbiome in MMVD and HF. He emphasizes that systems biology and high-throughput multi-omics techniques are likely to be used for canine MMVD and HF, and that as new techniques emerge, it will be possible to provide breakthrough nutritional interventions for the treatment of pet dogs with heart disease such as MMVD.

Therefore, the Research Topic provides a review of the articles on the nutritional management of animal nutrition metabolism, and lists some of the authors' new research inspirations based on the current research results and some of the significant contributions of the researches.

## Author Contributions

All authors listed have made a substantial, direct, and intellectual contribution to the work and approved it for publication.

## Funding

The National Key R&D Program (2021YFD1301004, 2021YFD1301005, and 2021YFD1300401) and China Agriculture Research System of MOF and MARA.

## Conflict of Interest

The authors declare that the research was conducted in the absence of any commercial or financial relationships that could be construed as a potential conflict of interest.

## Publisher's Note

All claims expressed in this article are solely those of the authors and do not necessarily represent those of their affiliated organizations, or those of the publisher, the editors and the reviewers. Any product that may be evaluated in this article, or claim that may be made by its manufacturer, is not guaranteed or endorsed by the publisher.
